# C-tag TNF: a reporter system to study TNF shedding

**DOI:** 10.1074/jbc.RA120.015248

**Published:** 2021-01-13

**Authors:** Francesca Pinci, Moritz M. Gaidt, Christophe Jung, Gunnar Kuut, Margaret A. Jackson, Stefan Bauernfried, Veit Hornung

**Affiliations:** Gene Center and Department of Biochemistry, Ludwig-Maximilians-Universität München, Munich, Germany

**Keywords:** Tumor necrosis factor, ADAM, shedding, CRISPR/Cas, reporter, cell surface enzyme, microscopy, flow cytometry, tumor necrosis factor (TNF)

## Abstract

TNF is a highly pro-inflammatory cytokine that contributes not only to the regulation of immune responses but also to the development of severe inflammatory diseases. TNF is synthesized as a transmembrane protein, which is further matured via proteolytic cleavage by metalloproteases such as ADAM17, a process known as shedding. At present, TNF is mainly detected by measuring the precursor or the mature cytokine of bulk cell populations by techniques such as ELISA or immunoblotting. However, these methods do not provide information on the exact timing and extent of TNF cleavage at single-cell resolution and they do not allow the live visualization of shedding events. Here, we generated C-tag TNF as a genetically encoded reporter to study TNF shedding at the single-cell level. The functionality of the C-tag TNF reporter is based on the exposure of a cryptic epitope on the C terminus of the transmembrane portion of pro-TNF on cleavage. In both denatured and nondenatured samples, this epitope can be detected by a nanobody in a highly sensitive and specific manner only upon TNF shedding. As such, C-tag TNF can successfully be used for the detection of TNF cleavage in flow cytometry and live-cell imaging applications. We furthermore demonstrate its applicability in a forward genetic screen geared toward the identification of genetic regulators of TNF maturation. In summary, the C-tag TNF reporter can be employed to gain novel insights into the complex regulation of ADAM-dependent TNF shedding.

TNF (Tumor necrosis factor) is a pro-inflammatory cytokine involved in the regulation of immune responses, inflammation, and cell death (1, 2). The main source of TNF are macrophages, but it can also be released by other cell types, including T and B lymphocytes, neutrophils, and fibroblasts, albeit at a lower level (1). Dysregulated TNF signaling, either via aberrant production or abnormal activation of its receptors TNFR1 and TNFR2, is associated with severe inflammatory diseases, such as rheumatoid arthritis, inflammatory bowel disease and many other pathological conditions (2, 3). In fact, the central role of TNF in coordinating inflammatory responses is well documented by its successful targeting in various disease entities (2, 3).

TNF is produced as a type II transmembrane protein that assembles into trimers on the cell membrane. To be released, TNF needs to be cleaved by membrane metalloproteases, mainly ADAM17 (A disintegrin and metalloprotease 17) (4, 5) but also, yet to a minor extent, by other family members, such as ADAM10 (6, 7, 8, 9). ADAM proteases are involved in the cleavage of numerous proteins from the cell membrane, thereby contributing to many biological processes, such as cell adhesion and migration, signaling, and immune responses (10, 11). Because of the high number of substrates and the irreversibility of the proteolytical cleavage as a post-translational modification, the activity of ADAM proteases is controlled by the cell at different levels. ADAM17, firstly discovered as the TNF shedding enzyme (Tumor necrosis factor alpha converting enzyme, TACE) (4, 5) is the best characterized enzyme of the ADAM family and its regulation has been extensively investigated (10, 12). One of the first discovered regulatory mechanisms of ADAM17 is the inhibition of the catalytic domain by its pro-domain to prevent unspecific cleavage of proteins on the way to the membrane (10). The pro-domain is then cleaved in the Golgi by convertases such as FURIN, to render the enzyme active (12, 13). Other mechanisms of regulation include phosphorylation of the cytosolic tail, interactions with phospholipids on the cell membrane to promote the interaction of the catalytic domain with its substrates, inhibition of the catalytic domain by the extracellular TIMP proteins and modifications by PDI enzymes (12). Moreover, rhomboid-like iRhom proteins (iRhom1 and iRhom2) are essential to govern the maturation and activity of ADAM17, mainly by contributing to its stability on the cell membrane, but also by other more recently described mechanisms (14, 15, 16). Because of their role in multiple physiological and pathological aspects, TNF and its sheddase ADAM17 have been intensively studied. However, the complexity of their function and regulation still requires further research efforts.

TNF production is typically assessed by end point analysis techniques of bulk cell cultures or tissues, detecting the matured version of the cytokine. Although well established, these approaches do not provide single cell resolution, which precludes them from being employed in genetic screens or kinetic analyses of TNF production at the cellular level. Flow cytometry or immunofluorescence microscopy techniques, on the other hand, can be used to determine pro-TNF abundance at the single cell level, yet they cannot inform on the maturation status of the cytokine. Moreover, relying on the fixation and permeabilization of cells, these techniques are disruptive. To circumvent these limitations, we here generated a genetically encoded reporter system that allows the study of the dynamics of TNF shedding at single cell resolution.

## Results

### The C-tag TNF is cleaved in a similar fashion as WT TNF in HEK293T cells

**Figure 1:**
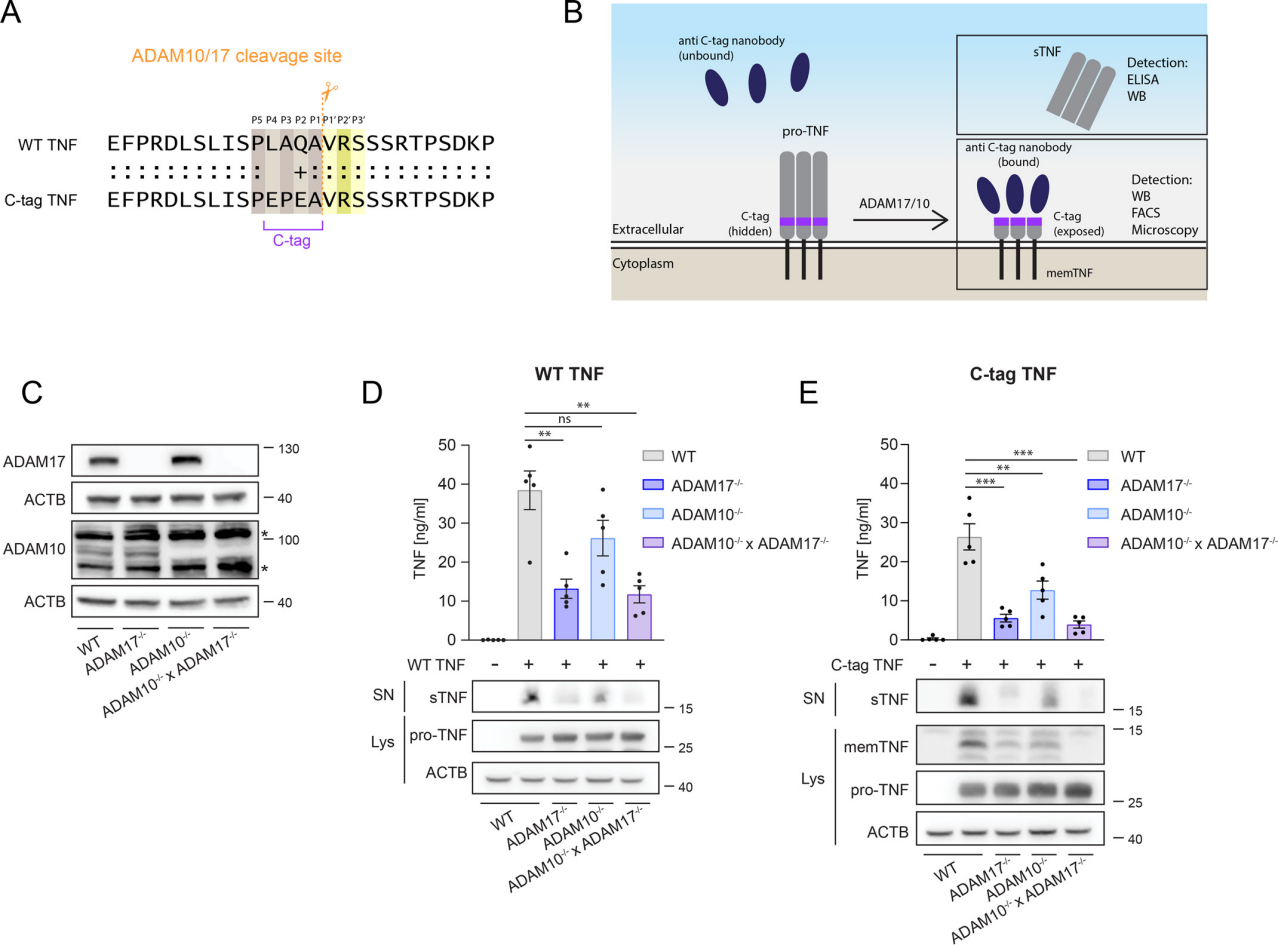
**C-tag TNF is shed in a similar fashion as WT TNF in HEK293T cells.***A*, Schematic overview of the C-tag TNF reporter. The C-tag is inserted in the TNF sequence before the ADAM17/10 cleavage site (between A76 and V77). Colons (:) represent identical amino acids, the plus sign (+) indicates a similar amino acid. *B*, Overview of possible detection methods for the C-tag TNF reporter. *C*, Immunoblotting of ADAM17 and ADAM10 in HEK293T cells of the indicated genotypes. * marks unspecific bands. *D*–*E*, HEK293T cells of the indicated genotypes were transfected with a control vector (pLI_mCherry) or with pLI_TNF (*D*) or pLI_C-tag TNF (*E*). Twenty-four hours after transfections, the expression of WT TNF and C-tag TNF was induced with doxycycline (1 µg/ml) in 2% FCS containing medium for 7 h before harvesting. Samples were analyzed via TNF ELISA and immunoblotting. memTNF in (*E*) was detected in cell lysates by the anti-C-tag nanobody. Data are represented as means ± S.E.M of 5 independent experiment or as representative images of 4 independent experiments. Uncropped images of the depicted immunoblots can be found in Fig. S6.

Currently, detection of TNF mostly relies on ELISA or immunoblotting methods that analyze cell supernatants, tissue or serum samples for the cleaved, mature TNF. However, being a type II transmembrane protein, TNF also exposes a neo C terminus associated with the transmembrane region on cleavage. To detect TNF maturation at single cell resolution, we set out to design a reporter system in which the ADAM-cleaved, membrane-associated portion of TNF could be detected as a gain-of-function signal. Because, at least according to our knowledge, there is no antibody available that would only recognize the C-terminal portion of the membrane-associated, cleaved TNF, we focused on the approach to introduce a heterologous cleavage site that would generate a detectable neo-epitope on processing. To this end, we designed a genetically encoded TNF-based reporter in which we introduced the four amino acid C-tag (EPEA) directly before the characterized ADAM17 cleavage site (Fig. 1*A*, *B*). Introducing the C-tag at positions 73-76 of TNF creates a new recognition site, in which the naturally occurring positions P_4_, P_3_ and P_2_ are changed. However, the newly introduced amino acids are well compatible with the experimentally determined recognition sites of ADAM17 or ADAM10 (Fig. S1) (17). Because the C-tag can be recognized by a specific nanobody only if exposed at the C terminus of a protein (18), we hypothesized that the detection of the C-tag could occur only after TNF cleavage by ADAM proteases but not within the full-length TNF (Fig. 1*B*). To test this hypothesis, we set out to study the activation of the C-tag TNF reporter in HEK293T cells. Both ADAM17 and ADAM10, the two main sheddases of TNF, were found to be expressed in HEK293T cells (Fig. 1*C*). To address their involvement in TNF maturation, we generated ADAM17^−/−^, ADAM10^−/−^ and ADAM10^−/−^ × ADAM17^−/−^ HEK293T cells (Fig. 1*C*). Because HEK293T cells do not express TNF, we transiently transfected wild-type (WT) TNF and studied its shedding by ELISA and immunoblotting. As expected, TNF cleavage was mainly dependent on ADAM17, whereas ADAM10 contributed to a minor extent (Fig. 1*D*). We then analyzed the cleavage of C-tag TNF using the same approach. Soluble TNF levels (sTNF) in the supernatant of C-tag TNF transfected cells were slightly lower compared with what observed on transfection with WT TNF, suggesting that the C-tag TNF is a less efficient substrate for metalloproteases than its WT counterpart. However, C-tag TNF cleavage was also mainly ADAM17-dependent, and also showed a partial reduction in ADAM10-deficient cells (Fig. 1*E*). The N-terminal fragment of C-tag TNF (membrane bound TNF; memTNF) was detectable in the lysate of transfected WT HEK293T cells by immunoblotting using the C-tag nanobody. In line with the ELISA results, the C-tag and the sTNF signals were mainly dependent on ADAM17 and to a lesser extent on ADAM10 (Fig. 1*E*). Deletion of both ADAM17 and ADAM10 led to an almost complete loss in sTNF or C-tag signal. Further, introduction of the C-tag cleavage site did not interfere with pro-TNF binding to TNFR2 (Fig. S2). Taken together, these data show that WT TNF is mainly matured by ADAM17 and partially by ADAM10 in HEK293T cells. Moreover, the C-tag TNF reporter is cleaved in a similar manner as WT TNF in these cells. Importantly, the detection of the C-tag signal occurred specifically after maturation of TNF by metalloproteases.

### The C-tag TNF reporter as a tool in flow cytometry analysis of cleaved TNF

**Figure 2:**
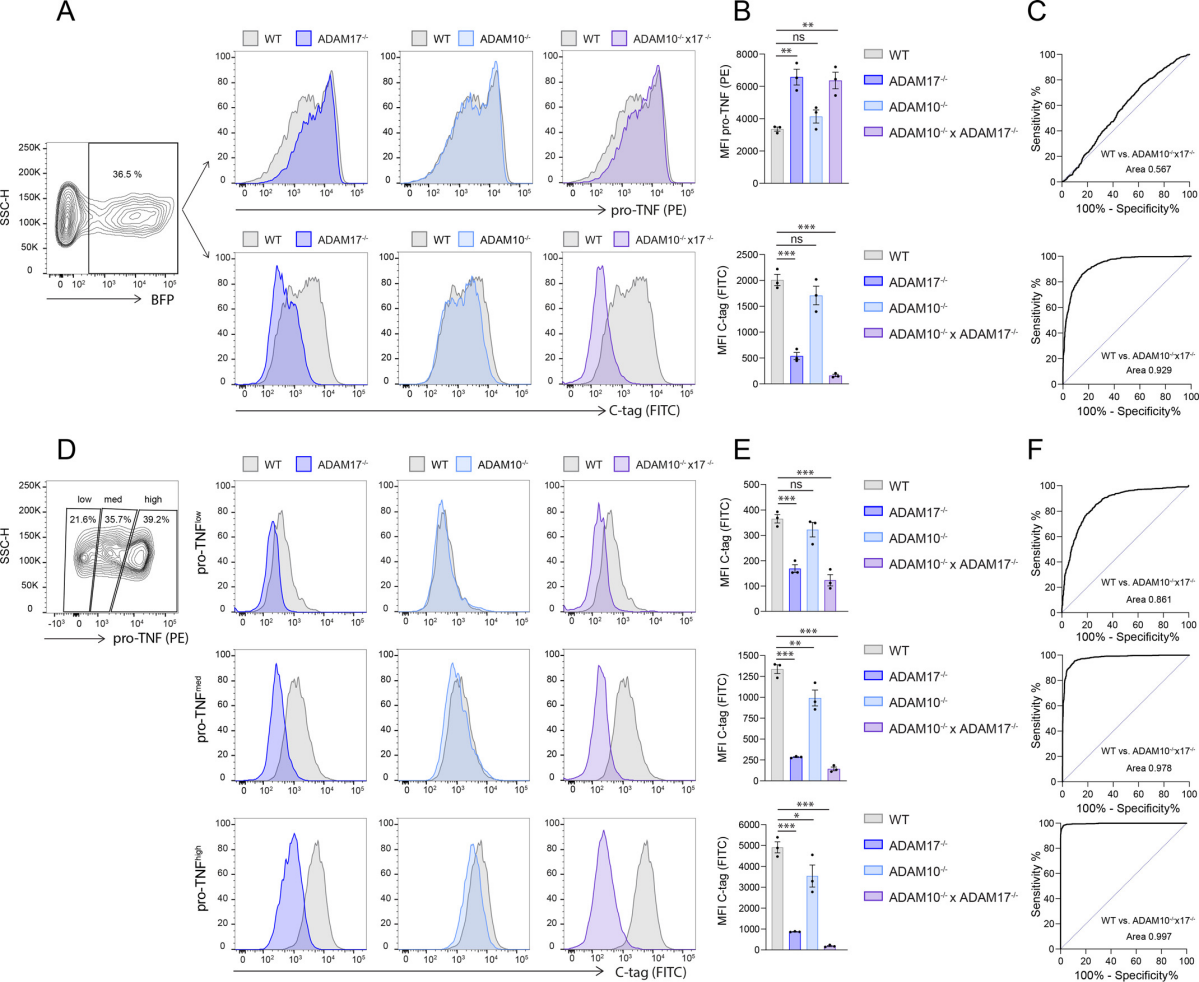
**The C-tag TNF reporter can be used to detect TNF shedding by flow cytometry.** HEK293T cells of indicated genotypes were transfected with pLI_BFP_C-tag TNF and protein expression induced by doxycycline for 36 h before analysis. *A*, Transfected cells were identified by BFP positivity and further analyzed for pro-TNF or C-tag positivity. *B*, Median fluorescence intensity (MFI) of pro-TNF and C-tag signals are reported in upper and lower panel, respectively. Data shown as means ± S.E.M of 3 independent experiments. *C*, ROC analysis of flow cytometry data shown in A to assess the discriminatory performance of pro-TNF and C-tag TNF staining between shedding and non-shedding (ADAM10^−/−^ × ADAM17^−/−^) cells. *D*, BFP positive cells were further separated in low, intermediate and high expression of pro-TNF before the analysis of C-tag signal in WT, ADAM17^−/−^, ADAM10^−/−^ and ADAM10^−/−^ × ADAM17^−/−^ cells. *E*, MFI analysis of C-tag signal of histograms depicted in (D). Data shown as means ± S.E.M of 3 independent experiments. *F*, ROC analysis of flow cytometry data shown in (*D*) (upper, middle. and lower panel, respectively). Flow cytometry plots in (*A*) and (*D*) are representative of 3 independent experiments.

The maturation of C-tag TNF by ADAM metalloproteases leaves the C-tag accessible to its specific nanobody on the outer membrane of the cells. To investigate whether the C-tag TNF reporter could be employed in flow cytometry experiments, we transfected WT, ADAM17^−/−^, ADAM10^−/−^ and ADAM10^−/−^ × ADAM17^−/−^ HEK293T cells with a plasmid that encodes for the C-tag TNF reporter and for a blue fluorescent protein (BFP) (Fig. 2*A*). Gating on BFP-positive cells, we first employed an antibody that detects pro-TNF and assessed whether ADAM deficiency would increase the pro-TNF signal in these cells. Comparing cells of different ADAM genotypes for their pro-TNF expression level, increased pro-TNF levels were indeed observed for ADAM17^−/−^ and ADAM10^−/−^ × ADAM17^−/−^ cells (Fig. 2*A* and *B*, upper panel). However, the individual populations displayed overlapping peaks, so that they could not be clearly separated from one another. We assessed the discriminatory performance of the pro-TNF fluorescence intensity to separate TNF shedding cells from non-shedding cells, as determined by their ADAM genotype, using a receiver operating characteristic (ROC) analysis (Fig. 2*C*, upper panel). This analysis confirmed the notion that determining pro-TNF levels was not suited to separate shedding from non-shedding cells (ROC area under curve (AUC) = 0.567). However, using the C-tag nanobody to identify cells for TNF maturation, a much better separation of the different cell populations was achieved (Fig. 2*A*–*C*, lower panel). WT cells displayed a strong C-tag signal and this signal was decreased in ADAM17^−/−^ cells, indicating a lack of TNF processing (Fig. 2*A* and *B*, lower panel). ADAM10 deficiency on its own only showed a slight phenotype, yet its contribution to TNF maturation was more evident in ADAM10^−/−^ × ADAM17^−/−^ cells that showed almost half the C-tag TNF signal compared with ADAM17^−/−^ cells. Indeed, the discriminatory performance of the C-tag signal to separate shedding from non-shedding cells (WT *versus* ADAM10^−/−^ × ADAM17^−/−^ cells), as determined by ROC analysis, was much better compared with measuring pro-TNF levels (ROC AUC = 0.929) (Fig. 2*C*, lower panel). To further enhance the discrimination of cells that have cleaved TNF, we additionally stained pro-TNF on the cell surface. Based on their pro-TNF expression levels, we separated pro-TNF^low^, pro-TNF^med^ and pro-TNF^high^ cells (Fig. 2*D*–*F*). Analyzing the C-tag positivity as a function of their genotype, the best separation of WT cells from all other genotypes was observed for the pro-TNF^high^ population (Fig. 2*D*–*E*, lower panel). In particular, the peaks of cleaved TNF (C-tag signal) of WT and ADAM10^−/−^ × ADAM17^−/−^ cells were almost completely separated, with a ROC AUC of 0.997 (Fig. 2*F*, lower panel). Again, a stronger contribution of ADAM17 and a redundancy with ADAM10 was observed. At lower levels of pro-TNF expression, the discriminatory capacity of WT *versus* ADAMs-deficient cells by C-tag positivity decreased (Fig. 2*D*–*F*, upper and middle panel). Of note, the residual C-tag signal observed in ADAM10^−/−^ × ADAM17^−/−^ cells is most probably attributable to TNF shedding by other proteases. As such, the expression of an uncleavable C-tag TNF reduced the C-tag signal to background levels (Fig. S3). In summary, these results indicate that the C-tag TNF reporter can be employed in flow cytometry analysis experiments alone or in combination with pro-TNF staining, enhancing the ability to discern between shedding and non-shedding cells.

### Live cell imaging analysis of C-tag TNF shedding

**Figure 3:**
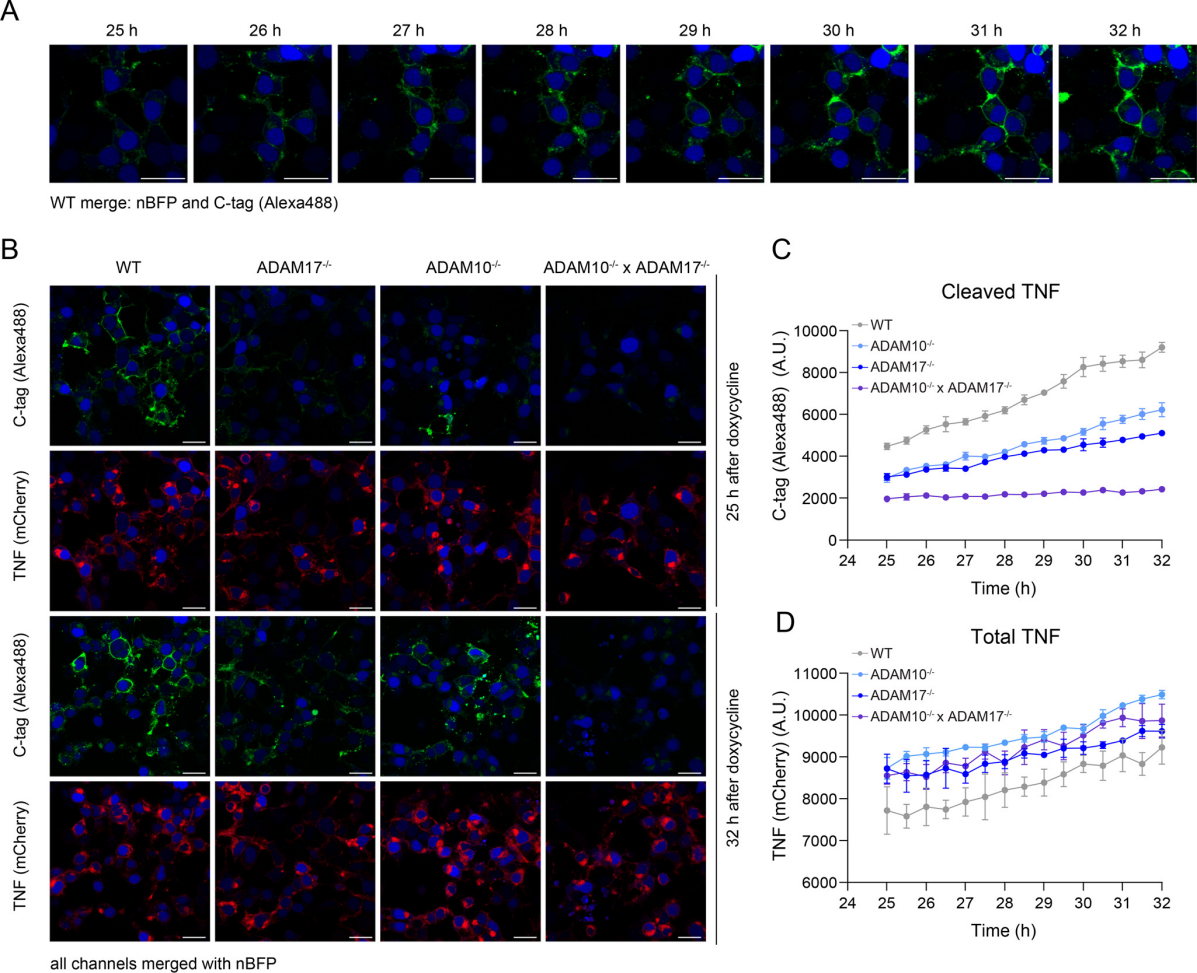
**Live cell imaging of C-tag TNF shedding by confocal microscopy.** HEK293T cells of the indicated genotypes were transiently transfected with pLI_C-tag TNF linker mCherry and pEF-BOS_nBFP, treated with doxycycline (1 µg/ml) for 25 h and imaged via confocal microscopy for a period of 7 h. *A*, Increasing positivity for C-tag TNF observed over time in WT HEK293T cells. Green = Alexa488 anti C-tag, blue = nBFP (nuclear BFP). *White bar* = 25 µm. *B*, C-tag and TNF linker mCherry signals observed over time by live cell imaging in cells of indicated genotypes. *White bar* = 25 µm. *C*–*D*, Quantification of cleaved TNF (*C*) and total TNF (*D*), respectively corresponding to Alexa488 anti-C-tag nanobody and mCherry signals. For each independent experiment, the average of signals from single cells in each channel was calculated. Data are presented as mean ± S.E.M of three independent experiments.

To further explore the range of applications of the C-tag TNF reporter, we tested the possibility to visualize TNF shedding over time by live cell imaging. For this purpose, we generated a variant of the C-tag TNF reporter bearing an mCherry tag linked to its cytosolic tail (C-tag TNF linker mCherry). This way, both the total amount of TNF as well as cleaved TNF can be visualized at the same time during the analysis. Transfecting cells with this reporter, we conducted a series of live cell imaging studies of WT cells by confocal microscopy over a period of 7 h. These studies revealed that TNF shedding can be monitored on addition of the fluorescently labeled anti-C-tag nanobody to the medium (Fig. 3*A*, Movie S1). The accumulation of cleaved TNF on shedding allows the anti-C-tag nanobody to bind to its target, leading to a gain of fluorescent signal at the cell membrane (Fig. 3*A*). Importantly, the C-tag positivity depended on the presence of ADAM proteases, as demonstrated by a diminished fluorescent signal observed in ADAM17^−/−^, ADAM10^−/−^ and, most strikingly, in ADAM10^−/−^ × ADAM17^−/−^ cells (Fig. 3*B* and *C*). These results are consistent with data obtained by ELISA, immunoblotting and flow cytometry (Figure 1, Figure 2). Of note, no significant difference was observed for the mCherry signal among the genotypes, thereby excluding that differential expression of pro-TNF would account for a difference in fluorescence intensity of the C-tag-reporter (Fig. 3*C* and *D*). In summary, these data demonstrate that the C-tag reporter can be utilized in live cell imaging experiments to follow ADAM proteases-dependent shedding events over time.

### A CRISPR/Cas9 genome-wide FACS-based screen with the C-tag TNF reporter

**Figure 4:**
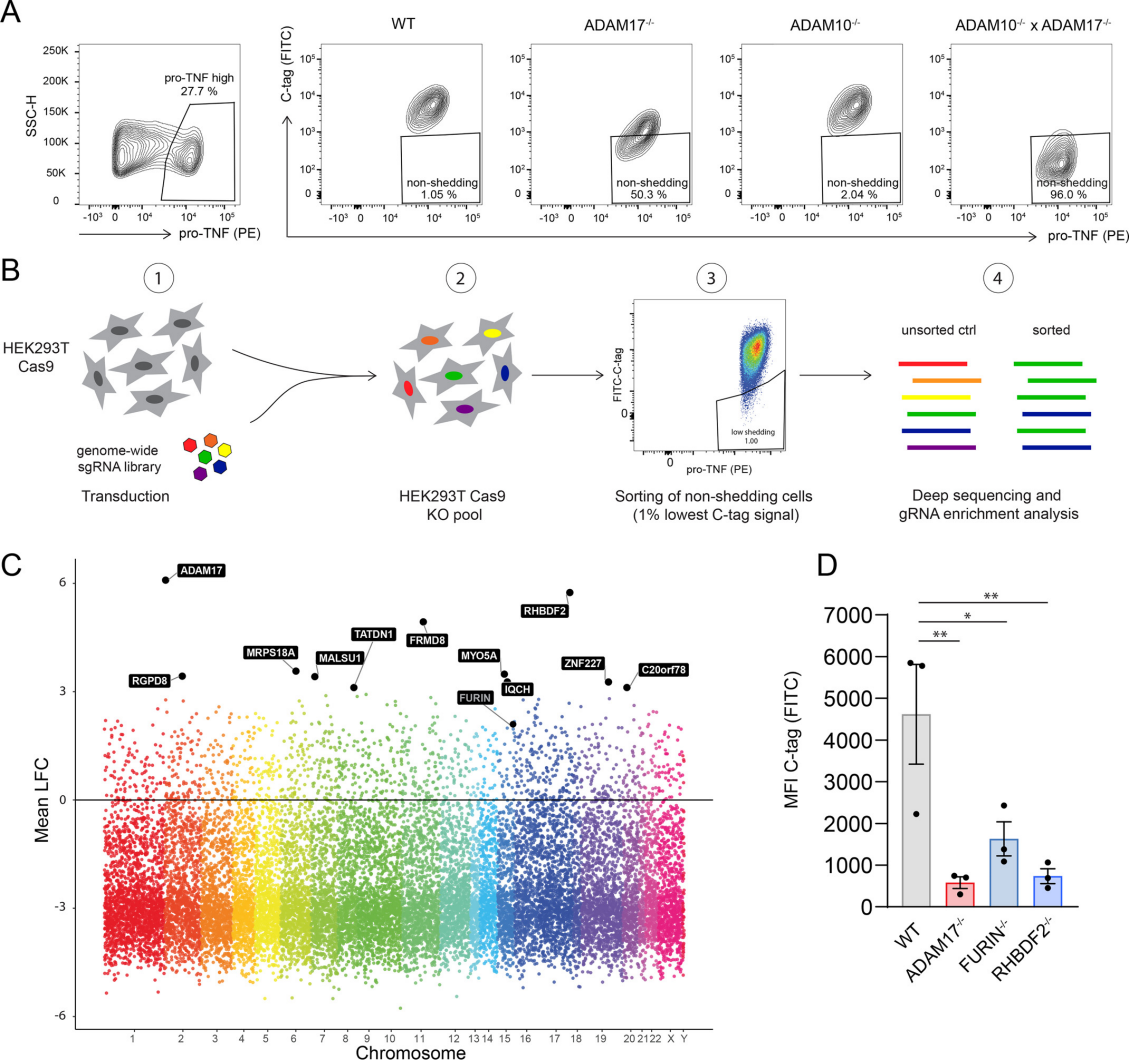
**A CRISPR/Cas9 genome-wide screen with the C-tag TNF reporter allows the identification of genetic factors involved in TNF shedding.***A*, HEK293T cells of depicted genotypes were transfected with pLI_C-tag TNF and protein expression induced for 36 h before analysis. Cells were pre-gated for high pro-TNF expression and then plotted for pro-TNF and C-tag TNF expression. A non-shedding population was identified by comparing the C-tag signal of WT cells with the signal obtained from ADAM17^−/−^, ADAM10^−/−^ and ADAM10^−/−^ × ADAM17^−/−^ cells. One representative of 3 independent experiments. *B*, Schematic overview of the CRISPR/Cas9 screen strategy. *C*, Manhattan plot of hits obtained from the genetic screen. Data are shown as log2 of mean fold change (Mean LFC) of all gene specific gRNAs (max 4 gRNAs/gene) compared with unsorted control. The name of hits with mean LFC > 3 from the gene ranking list generated by PinAPL-Py analysis of screening data are reported. Additionally, the known regulator FURIN is depicted in the figure. *D*, WT, FURIN^−/−^ and RHBDF2^−/−^ cells were transfected and doxycycline-treated as in (*A*). Cells were pre-gated on pro-TNF high expression and were further analyzed for C-tag signal. MFI of C-tag signal from pro-TNF high population is shown. Mean ± S.E.M of 3 independent experiments. One representative of 2 independent clones per genotype.

In light of the ability to separate WT from ADAM10^−/−^ × ADAM17^−/−^ cells by C-tag TNF positivity at the single cell level, we set out to conduct a forward genetic screen to identify factors required for TNF shedding. To do so, we stained WT, ADAM17^−/−^, ADAM10^−/−^ and ADAM10^−/−^ × ADAM17^−/−^ cells for pro-TNF and the C-tag and devised a gating strategy to separate shedding from non-shedding cells (Fig. 4*A*). With this gating, 96% of the ADAM10^−/−^ × ADAM17^−/−^ cell line could be identified as non-shedding cells. We generated HEK293T cells stably expressing Cas9 and transduced these cells with a genome-wide gRNA lentiviral library. To avoid multiple gRNA integrations per target cell, we infected cells with a multiplicity of infection (MOI) of 0.3. After antibiotic resistance selection and expansion, successfully transduced cells were transiently transfected with the C-tag TNF construct and protein expression induced by doxycycline treatment. On the day of sorting, a fraction of unsorted cells was kept as a negative control and the rest were stained for pro-TNF and C-tag. On FACS analysis, we pre-gated on pro-TNF high cells (about 18% of total single cells) and subsequently sorted for non-shedding cells as identified as cells with a low C-tag signal (Fig. 4*B* and S^4^*A*). This resulted in the selection of ∼1% of cells of the parental cell population. Next, we performed deep sequencing of unsorted control and sorted non-shedding cells to identify gRNAs enriched in the non-shedding population. 98.7% of the original gRNAs were represented in our unsorted control population, confirming a very high representation of the library in our screen (Fig. S4B). Using PinAPL-Py (19), we generated a gene ranking list with the most targeted genes in the non-shedding population (Fig. 4*C*, Table S1). This analysis considered both the number of gRNAs found to target a specific gene (with a maximum of 4 gRNAs) and the level of enrichment of each individual gRNA compared with the unsorted control. According to this analysis, the first-ranked hit was ADAM17, immediately followed by RHBDF2 (iRhom2), confirming the validity of our screening approach. Moreover, as the third-ranked hit we found FRMD8, which was recently described to be involved in the stability of ADAM17/iRhom2 complex on the cell membrane (20, 21). In addition, FURIN deficient cells were also enriched in the non-shedding population, even if not as strongly as the other known regulators. We confirmed the ability to detect RHBDF2 and FURIN deficient cells with our flow cytometry approach by generating RHBDF2^−/−^ and FURIN^−/−^ clones in HEK293T cells and analyzing them for their C-tag signal (Fig. 4*D*). Although RHBDF2^−/−^ cells phenocopied the deficiency for ADAM17, FURIN^−/−^ cells exhibited a less pronounced reduction of their C-tag signal, in line with the more modest enrichment of FURIN targeting gRNAs in the non-shedding population observed in the screen. Of note, ADAM10 was not ranked as a hit in our screen, consistent with the minor reduction of C-tag signal observed for ADAM10^−/−^ cells in previous experiments. Next, we conducted a STRING analysis to retrieve potential protein-protein interactions within the group of proteins that were scored as hits, as defined by an enrichment of their mean fold change >2 (22). Doing so, we identified additional, potential regulators of ADAM17-dependent TNF shedding (Fig. S5). AP2M1, which encodes for a component of the AP-2 complex involved in protein transport in membrane traffic pathways, was identified as a positive regulator being associated with FURIN. An involvement of AP2M1 is in so far conceivable, as previous studies show AP2M1 and AP2 as direct interactors of FURIN and ADAM10, respectively (23, 24). Moreover, the metalloprotease ADAMTS1 (a disintegrin and metalloproteinase with thrombospondin motifs 1) was identified within the data set as another potential interactor of FURIN, suggesting a potential role for ADAMTS1 in TNF shedding in our settings. Taken together, the detection of the C-tag TNF reporter by flow cytometry could be used with success in a CRISPR/Cas9-based screen for the identification of genetic factors involved in TNF shedding.

## Discussion

In this study, we generated and validated a new reporter system (C-tag TNF) for the detection of TNF cleavage. This reporter exploits the introduction of the C-tag as a cryptic epitope at the TNF cleavage site of ADAM17. On exposure of the membrane portion of pro-TNF that is generated on shedding, the C-tag epitope is efficiently and specifically detected by a nanobody. We could demonstrate that this reporter is compatible with different techniques, namely ELISA, immunoblotting, flow cytometry and live cell imaging. The cleavage of WT and C-tag TNF was mainly dependent on ADAM17, whereas ADAM10 showed only a minor impact on TNF maturation. Residual TNF shedding could be detected in the ADAM10^−/−^ × ADAM17^−/−^ cells at different levels depending on the technique employed. End point techniques such as ELISA and immunoblotting showed a higher background in terms of ADAM10/17-independent TNF maturation. This is likely attributable to the fact that these techniques measure cumulative TNF levels over the entire assay and at the same time, these assays also detect soluble TNF that has been matured at a different cleavage site. In flow cytometry and live cell imaging experiments, only the specific C-terminal fragment of the cleaved TNF construct that is present at a specific time point on the cell membrane is responsible for the shedding-specific signal. Thereby the background of ADAM10/17-independent TNF shedding is reduced. Nevertheless, even under these conditions, a slight, ADAM10/17-independent cleavage signal of TNF was observed.

The ability to monitor the shedding activity by flow cytometry and live cell imaging enables the investigation of shedding events at single-cell resolution in real time. Indeed, the C-tag signal was superior to assessing the pro-TNF signal in its ability to separate WT cells from ADAM17^−/−^ and ADAM10^−/−^ × ADAM17^−/−^ deficient cells in flow cytometry experiments. This discriminatory capacity allowed us to perform a FACS-based genetic screen for the identification of factors involved in TNF shedding. The top ranked hits of the screen were known regulators of TNF shedding, namely ADAM17, RHBDF2 and FRMD8, confirming the validity of our approach. In addition, a STRING analysis highlighted other potential regulators of TNF shedding, in particular a factor involved in vesicle trafficking (AP2M1) and another metalloprotease (ADAMTS1). However, further studies are required to investigate their involvement in TNF maturation.

At present, TNF shedding is often indirectly studied by quantification of the mature form of the cytokine in bulk cell cultures or tissues. Despite the existence of excellent anti-TNF antibodies that can be reliably employed in bulk techniques, these often only allow for an end point quantification of the amount of pro-TNF and matured TNF in a certain sample, but do not contribute to detecting the interaction between the enzyme and the substrate or the shedding event itself. The same limitation applies to alkaline phosphatase (AP) reporters, where AP, fused to the extracellular domain of the substrate under investigation, is released in the supernatant on cleavage by membrane metalloproteases (25). In the attempt to overcome this limitation and to focus the attention on the shedding process rather than on the quantification of the mature substrate, a number of assays have been developed over the years. Fluorogenic peptides, bearing the cleavage site of a specific ADAM with a fluorophore and a quencher at the opposite sides of the peptide chain, have been generated to measure ADAM protease activity by fluorescence-based methods (26). However, the cleavage of a small peptide by ADAM proteins can considerably differ from the ability of the proteases to cleave their specific substrate on the cell membrane. In fact, the accessibility to the substrate is governed not only by the availability of the catalytic domain of the enzyme, but also by the reciprocal positioning of the substrate and the ADAM protein itself. Additional reporter systems have been generated to study other membrane-associated proteases. For example, a reporter based on the amyloid precursor protein (APP) has been designed to detect the amount of cleaved APP, by quantification of AP activity in the supernatant, and simultaneously the interaction between APP and its protease Beta-secretase 1, employing a luciferase complementation assay (27). Moreover, FRET-based reporters of APP have been described (28, 29).

The C-Tag TNF reporter can be employed to visualize TNF shedding at single cell level in living cells. Moreover, the C-tag TNF reporter has additional advantages compared with previously described methods for the detection of TNF maturation or ADAM activity. First, it requires little genetic manipulation. Only three amino acids need to be exchanged in the WT TNF sequence, whereas there is no need to introduce additional functional domains (*e.g.* fluorescent colors or enzymes) that could impact the shedding process. In addition, because the C-tag is retained on the membrane fragment of pro-TNF, its shedding leaves the mature, soluble cytokine untouched. This is of importance if the activity of the shed TNF needs to be retained. Of note, the uncleaved C-tag TNF also retains its binding capacity to TNFR2, which allows the employment of this reporter in studies where the effects of both soluble and membrane-bound TNF are investigated. In light of their relaxed cleavage site specificities, we speculate that the C-tag would also be compatible with other metalloproteases, which would make this approach a general blueprint for generating reporter systems for these enzymes. However, it should be considered that this approach is only applicable to substrates that expose a free C terminus on the cell membrane after cleavage.

In summary, the C-tag TNF reporter represents a new tool that can be employed to elucidate the mechanisms of TNF shedding. Understanding the molecular details that contribute to the fine regulation of this process can have a decisive impact on our knowledge at the basis of TNF- and ADAM17-related diseases and on the development of new therapeutic approaches to modulate this pathway.

## Experimental Procedures

### Cell culture

HEK293T cells were cultured in DMEM Medium (Life Technologies) supplemented with 10% FCS (v/v), 100 U penicillin-streptomycin and 1 mm sodium pyruvate (all Life Technologies). Cells were split every 2–3 days or on plating.

### Transfections and stimulations

HEK293T cells of indicated genotypes were transfected in different formats and with different plasmids according to the experimental need. Transfections were always performed with GeneJuice transfection reagent (Merck Millipore) according to manufacturer's instructions. Doxycycline (Sigma-Aldrich) was always used at the final concentration of 1 μg/ml. For immunoblotting and ELISA experiments, 0.7 × 10^6^ cells were plated in each well of a 6-well plate and transfected the day after with 3 μg of the respective plasmid (pLI_C-tag TNF, pLI_TNF or pLI_mCherry). Twenty-four hours after transfection, cells were treated with doxycycline in 2% FCS containing medium for 7 h before harvesting. For flow cytometry analysis, 0.2 × 10^6^ cells were plated in 24-wells and transfected the day after with 800 ng of the indicated plasmid (pLI_BFP_C-tag TNF). Cells were treated with doxycycline for 36 h before the analysis. For confocal microscopy, 2.5 × 10^4^ cells were plated in collagen R (Serva) coated Ibidi 8-well u-Slides (Ibidi) using FluoroBrite DMEM medium (Gibco) supplemented as described above, with addition of 10 mm Hepes (Sigma-Aldrich). Cells were transfected with 50 ng each of pLI_C-tag TNF linker mCherry and pEF-BOS_nBFP for a total of 100 ng plasmid/well and treated with doxycycline at the same time. Twenty-five hours after transfection/induction, cells were analyzed via confocal microscopy as described.

### Genome-wide CRISPR/Cas9 screen

HEK293T cells stably expressing Cas9 were generated by transduction with the lentiviral construct lentiCas9-Blast (Addgene #52962, a gift from Feng Zhang) (30). Cells were selected with 10 μg/ml blasticidin (Thermo Fisher Scientific) and a monoclone with high Cas9 efficiency was used for further experiments; Cas9 activity was validated by a EGFP reporter assay (31). The human Brunello CRISPR knockout pooled library, a gift from David Root and John Doench (Addgene #73178) (32), was employed to perform the genome-wide CRISPR/Cas9 screen. A sample of 12 × 10^6^ cells were plated in each of 4 T175 flasks and infected with the gRNA library at a MOI of 0.3. The day after, virus was removed and cells were split into 12 T175 flasks. Twenty-four hours later, puromycin was added (3.3 μg/ml) to select for successfully infected cells. Four days later, 1.5 × 10^6^ cells were plated in 20 × 10 cm dishes and transfected the day after with 7.2 μg pLI_C-tag TNF plasmid/dish with GeneJuice. Seven hours after transfection cells were treated with 1 μg/ml doxycycline for 36 h. On the day of sorting, 300 × 10^6^ cells were detached from dishes, pooled and stained with CaptureSelect^TM^ biotin anti-C-tag conjugate (Thermo Fisher Scientific, 1:185) and PE anti-human TNF (MAb11, Biolegend, 1:70) for 30 min on ice. FITC-streptavidin (Biolegend) was employed to detect the anti-C-tag conjugate (1:100, incubation 30 min on ice). A sample of 20 × 10^6^ cells was left unstained and used as not sorted control. Both stained and not stained cells were fixed with 4% PFA for 10 min at room temperature. Cells were pre-gated for singlets with high PE-pro-TNF expression and ∼1% of cells with the lowest FITC-C-tag signal were sorted (non-shedding population) on a BD FACS Aria Fusion. Unsorted and sorted cells were then lysed as previously described (33) and lysate was used for PCR amplification of gRNA sequences with Phusion Polymerase (New England Biolabs). A nested PCR approach was employed to add Illumina sequencing adaptors, as previously described (33). For the first PCR, a mix of 8 different forward primers was used to ensure diversity of the deep sequencing library. Primer sequences can be found in Table S2.

### Sequencing and analysis

Samples were deep sequenced on an Illumina's Hi-Seq platform (HiSeq1500) with a 50 base single read. Data were analyzed by the online tool PinAPL-Py (19) for gRNAs enrichment in the non-shedding population compared with the unsorted control using default settings. Genes listed in the “gene ranking list” generated by the analysis, which considers both the number of gRNAs targeting a specific gene and their fold change of enrichment, were plotted according to chromosome position and LFC (log fold change) enrichment using R (ver 3.6). The top 10 enriched genes and the known regulator of ADAM17 function FURIN are highlighted in the plot.

### Immunoblotting

Protein precipitation from cell supernatants was performed with methanol/chloroform extraction. Cell pellets were lysed on ice in RIPA buffer containing a mixture of protease inhibitors (Roche). Quantification of proteins in cell lysates was performed via bicinchoninic acid (BCA) assay (Thermo Fisher Scientific). Cell lysates and protein precipitated from supernatant were denatured in Laemmli Buffer for 10 min at 95 °C before separation by denaturing SDS-PAGE. Equal amount of proteins from lysates (10 or 15 μg/lane) and equal volumes of precipitated supernatants (8-12 μl) were loaded in each lane. Proteins were transferred on 0.2 μm nitrocellulose membranes for 75 min at 100 V. After transfer, membranes were blocked in 5% milk and incubated with the indicated primary antibodies: TNF (D5G9, Cell Signaling Technology), ADAM17 (D22H4, Cell Signaling Technology), ADAM10 (AB19026, Merck Millipore), ACTB (sc-4778, Santa Cruz Biotechnology), CaptureSelect^TM^ biotin anti-C-tag conjugate (Thermo Fisher Scientific). Antibodies were used at 1:1000 dilution overnight, anti-C-tag conjugate was used at 1:800 dilution and incubated 36 h at 4 °C. Correspondent HRP-conjugated secondary antibodies (streptavidin-HRP in case of the biotin anti C-tag conjugate) were used for 1 h at room temperature to allow detection of chemiluminescent signals via a CCD camera (Fusion Fx, Vilber). When necessary, the contrast of images was enhanced in a linear fashion.

### Elisa

hTNF quantification in cell supernatant was performed with a hTNF ELISA kit (555212, BD Biosciences) following manufacturer's instruction.

### Flow cytometry analysis

HEK293T cells were trypsinized, washed once with FACS buffer (0.75% FCS, 2 mm EDTA) and stained with PE anti-human TNF (MAb11, Biolegend, 1:70) and CaptureSelect^TM^ biotin anti-C-tag conjugate (Thermo Fisher Scientific, 1:185) for 30 min on ice. After washing in FACS buffer, samples were stained with FITC-streptavidin (Biolegend) for 30 min on ice (1:100) to detect the anti-C-tag conjugate. Cells were subjected to a further wash before analysis on a BD LSR Fortessa. Flow cytometry data were analyzed using FlowJo 10.7. All histograms are shown normalized to mode.

### Plasmids

pLI and pLI_BFP are doxycycline-inducible plasmids. WT TNF, C-tag TNF and C-tag TNF linker mCherry were cloned into these plasmids by using the cloning sites NheI and BamHI. pEF-BOS was employed to express the nuclear TagBFP (nBFP) used in live cell imaging experiments, to facilitate cell identification on analysis. The nBFP sequence was inserted by employing XhoI and BamHI restriction sites.

### Receiver operating characteristic analysis

To determine the discriminatory capacity of either pro-TNF or C-tag staining to identify shedding or non-shedding cells, ROC curve analyses were conducted, comparing WT (determined as shedding cells) and ADAM10^−/−^ × ADAM17^−/−^ cells (determined as non-shedding cells). To this end, the individual fluorescence intensity values of 1000 randomly chosen events of each data set were obtained and a ROC curve analysis was conducted using GraphPad Prism 8. As a quantifiable measure, the area under the ROC curve is provided for each data set.

### Live cell imaging

Before imaging, the CaptureSelect^TM^ Alexa488 anti-C-tag conjugate (Thermo Fisher Scientific) was added to the cell supernatant at a final dilution of 1:480. Time series of confocal sections were acquired using a LEICA SP8 confocal microscope with a 40× (1.1 NA) water-immersion objective. Five regions of interest per genotype with a time resolution of one image per 30 min, over a period of 7 h, were scanned. Time series of confocal images were then processed by using the *Definiens XD 2.0* image analysis platform as follows. First, the contours of the nuclei were identified based on the nBFP signal in channel one. These contours were then used as seeds to identify the entire cell bodies based on the mCherry signal (total TNF) in channel 2. Finally, the average fluorescence intensity corresponding to the C-tag signal (Alexa488, channel 3) and the total TNF signal (mCherry, channel 2) was computed for each of the resulting segmented patterns. The analysis was performed for 4 regions of interest per genotype in each of 3 independent experiments. The average signal of all analyzed single cells from each replicate was computed and used to calculate the final mean ± S.E.M of 3 independent experiments.

### CRISPR/Cas9 knock-out and generation

Gene deficient HEK293T cells were generated as previously described. Briefly, gRNA specific for the genes of interest were selected from a genome-wide library (34). Cells were transfected with pLKO.1-gRNA-CMV-GFP and pRZ-BFP-Cas9 using GeneJuice. GFP and BFP double positive cells were sorted and subjected to limiting dilution cloning. After 3-4 weeks, monoclones were identified, transferred to a new 96-well plate and duplicated for genotyping by deep sequencing (Illumina Miseq platform). Clones with frameshift mutations were identified via Outknocker.org (33) and utilized for further experiments. If necessary, genotypes were further confirmed via Sanger sequencing. When indicated, protein depletion was verified via immunoblotting. Target sites of gRNAs and respective gene specific sequencing primers can be found in Table S2.

### Statistical analysis

Data were analyzed for statistically significant differences using One-way ANOVA with Dunnett's correction for multiple testing. The number of repetitions (n) is indicated in the Fig. legends. The analysis was performed with GraphPad Prism 8. *** *p* < 0.001, ** *p* < 0.01, * *p* < 0.05, ns= not significant.

## Data availability

Primary sequencing data from the genetic screen are available in the Sequence Read Archive (SRA) with the following accession ID: PRJNA644482.
