# Supplementary material for: C-tag TNF: a reporter system to study TNF shedding
Source: J Biol Chem. 2021 Jan 13;295(52):18065–75. doi: 10.1074/jbc.RA120.015248 (PMC7939438; doi:10.1074/jbc.RA120.015248)
Supplement: Supplementary file 1 [file mmc1.zip › 162303_2_supp_612277_qp700n.pdf]

Supporting Information

Supporting Legends

Supporting Movie S1. C-tag TNF shedding in HEK293T wild-type cells. Wild-type HEK293T cells were transiently transfected with pLI_C-tag TNF linker mCherry and pEF-BOS_nBFP, treated with doxycycline (1 µg/ml) for 25 h and imaged via confocal microscopy for a period of 7 h. Green = Alexa488 anti C-tag, blue = nBFP (nuclear BFP), white bar = 25 µm. The movie displays a representative experiment out of three independent experiments.

Supporting Table S1. Gene ranking list. Screening data were analyzed by the online tool PinAPL-Py for gRNA enrichment in the non-shedding population in comparison to unsorted control using default settings. Genes identified in the non-shedding population and listed in the "gene ranking list" are reported in the table with the average LFC (log fold change) and the number of targeting gRNAs that were enriched for each gene.

Supporting Table S2. Primer and gRNA sequences. Primer sequences for the analysis of the genome-wide CRISPR/Cas9 screen are shown. In addition, gRNA sequences for the individual gene targeting experiments as well as the respective genotyping primer sequences are provided.

Supporting Methods

Etanercept staining for flow cytometry analysis

HEK293T cells were transfected with the indicated plasmids and induced with doxycycline as described in the main experimental procedures. On the day of the analysis, cells were trypsinized, washed once with FACS buffer and incubated with 6 µg/ml Etanercept (Sigma-Aldrich) in 50 µl of FACS buffer for 30 minutes on ice. Cells were then washed with FACS buffer and stained with Alexa Fluor®647 anti-human IgG Fc (clone HP6017, Biolegend, 1:70) for 30 minutes on ice in the dark. Cells were subjected to a further wash before analysis on a BD LSR Fortessa. Samples were co-stained for C-tag positivity as described in the main section.

Uncleavable C-tag TNF

An uncleavable version of the C-tag TNF linker mCherry construct was generated by introducing the deletions of val77, val89 and the mutations Arg78Thr and Ser79Thr in the TNF sequence and expressed in the pLI inducible vector (pLI_C-tag TNF linker mCherry uncleavable mutant) (35,36). HEK293T cells of the indicated genotypes were transfected with pLI_C-tag TNF linker mCherry or the correspondent uncleavable mutant as described in the main session and assessed for C-tag TNF and pro-TNF expression by flow cytometry. C-tag staining was performed as already described. Pro-TNF was stained with the anti-TNF antibody D5G9, Cell Signaling Technology (1:100 in FACS buffer, 30 minutes on ice), followed by 30 minutes staining on ice with an Alexa488 anti-rabbit antibody (A11008, Life Technologies, used 1:100).
