# Supplementary material for: C-tag TNF: a reporter system to study TNF shedding
Source: J Biol Chem. 2021 Jan 13;295(52):18065–75. doi: 10.1074/jbc.RA120.015248 (PMC7939438; doi:10.1074/jbc.RA120.015248)
Supplement: Supplementary file 1 [file mmc1.zip › 162303_2_supp_614892_qqgtzl.pdf]

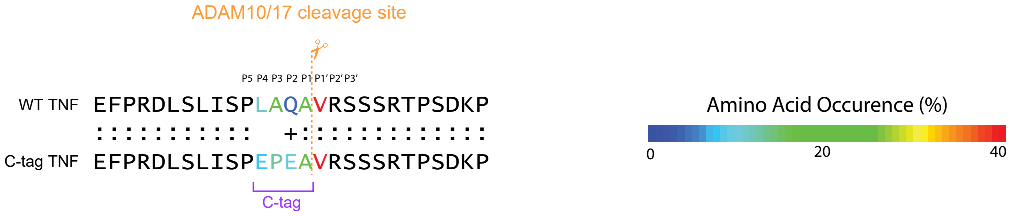

Figure S1. WT TNF and C-tag TNF sequences in the region of the cleavage site (related to Fig. 1). The amino acid sequences of the wildtype and C-tag TNF cleavage sites are shown. Colons (:) represent identical amino acids, the plus sign (+) indicates a similar amino acid. Colors indicate the amino acid occurrence (%) at the specific positions P4, P3, P2, P1 and P1' according to the previously, experimentally determined substrate specificity of ADAM17 (17).

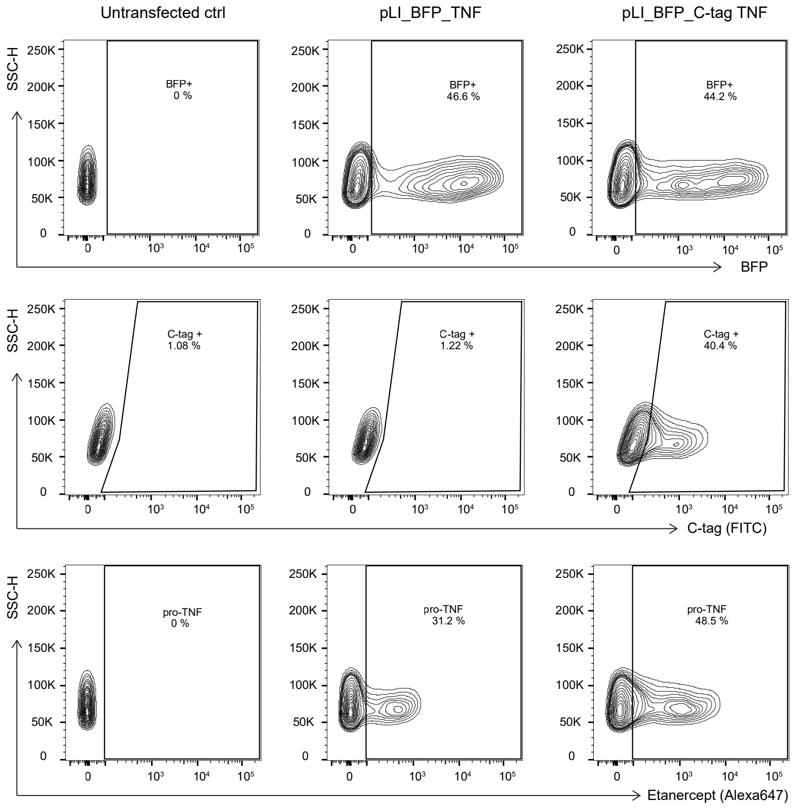

Figure S2. TNFR2 can bind to membrane-bound C-tag TNF. HEK293T cells were transfected with the indicated plasmids or left untransfected. Transfection efficiency was evaluated by BFP positivity (upper panel). Staining with FITC anti C-tag nanobody indicates the cleavage of TNF in cells transfected with pLI_BFP_C-tag TNF (middle panel). Staining with Etanercept indicates that both wt TNF and C-tag TNF can be bound by TNFR2 (lower panel). One representative of 2 independent experiments.

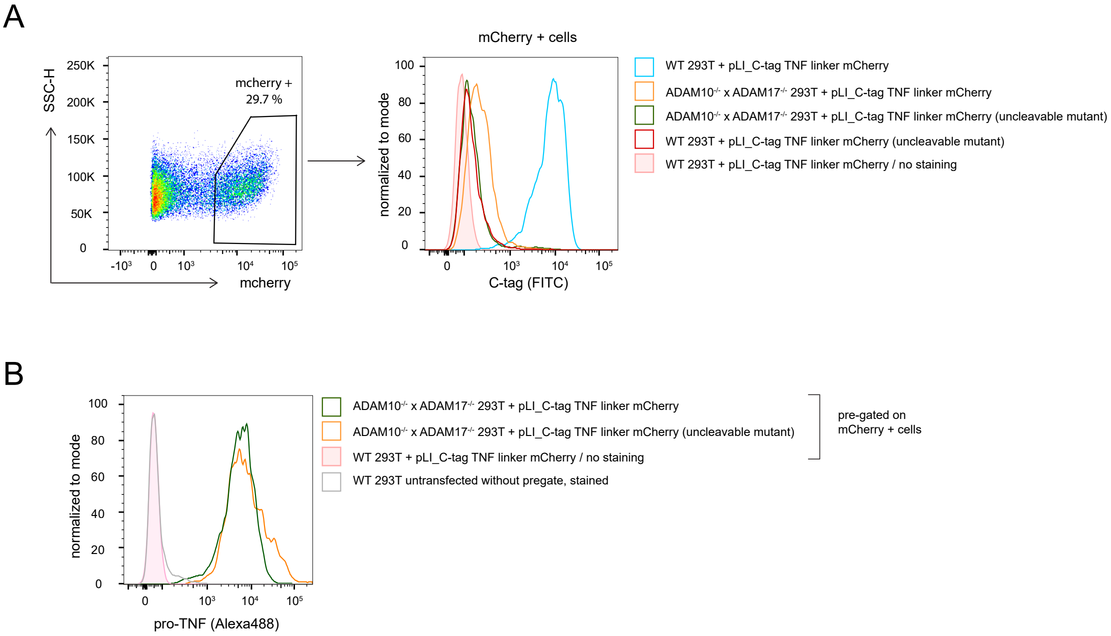

Figure S3. C-tag TNF residual shedding occurs independently of ADAM17 and ADAM10. HEK293T cells of the indicated genotypes were transfected with pLI_C-tag TNF linker mcherry or the correspondent uncleavable mutant and protein expression induced with doxycycline for 36 h before analysis. **A.** Cells were pre-gated for mCherry positivity and then further analyzed for C-tag expression. **B**. Cells transfected and doxycycline treated as described in A. were analyzed for pro-TNF expression on the cell membrane. The uncleavable C-tag TNF mutant was expressed on the cell membrane to the same extent as the regular C-tag TNF. One representative of two independent experiments is shown.

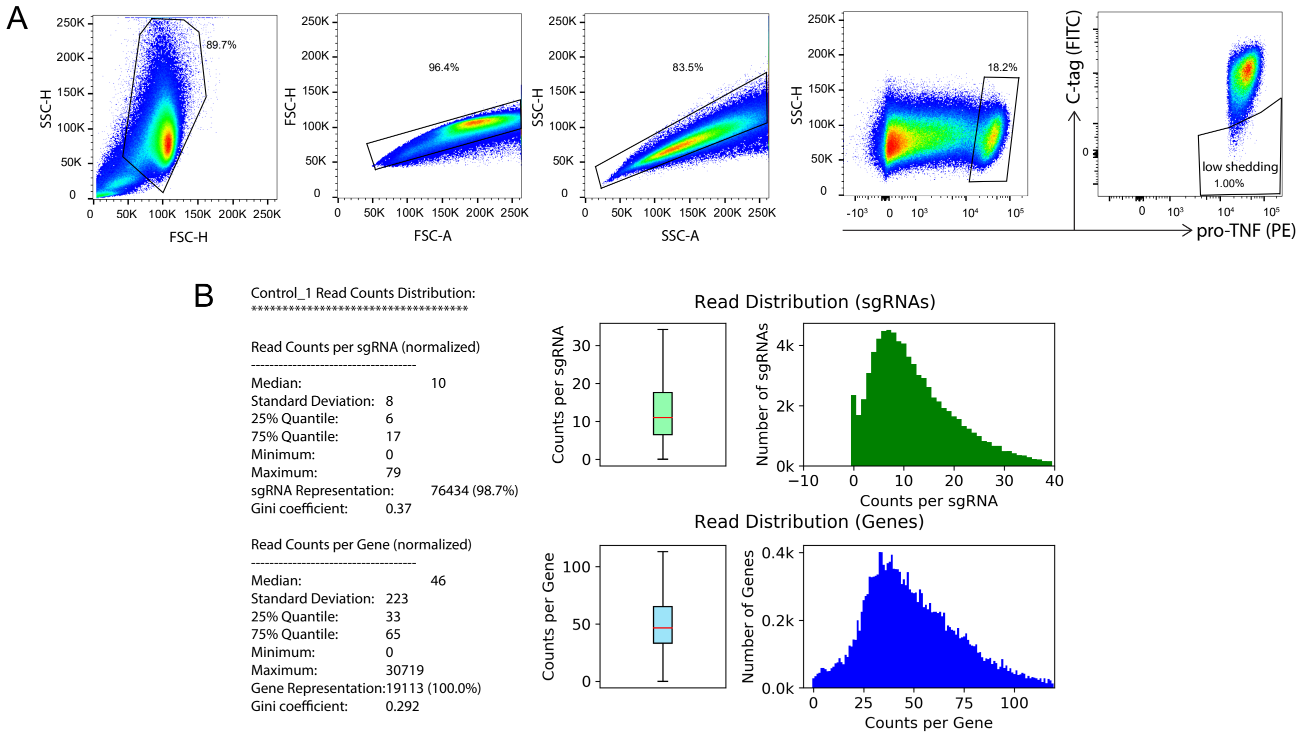

Figure S4. Sorting strategy and library representation of CRISPR/Cas9 genome-wide screen (related to Fig. 4). **A**. Sorting strategy of the non-shedding population on the day of sorting. **B**. Analysis of read distribution in the unsorted control sample. Data were obtained by analysis of deep sequencing data using the online tool PinAPL-Py. Read counts are provided as reads per 10^6 total reads.

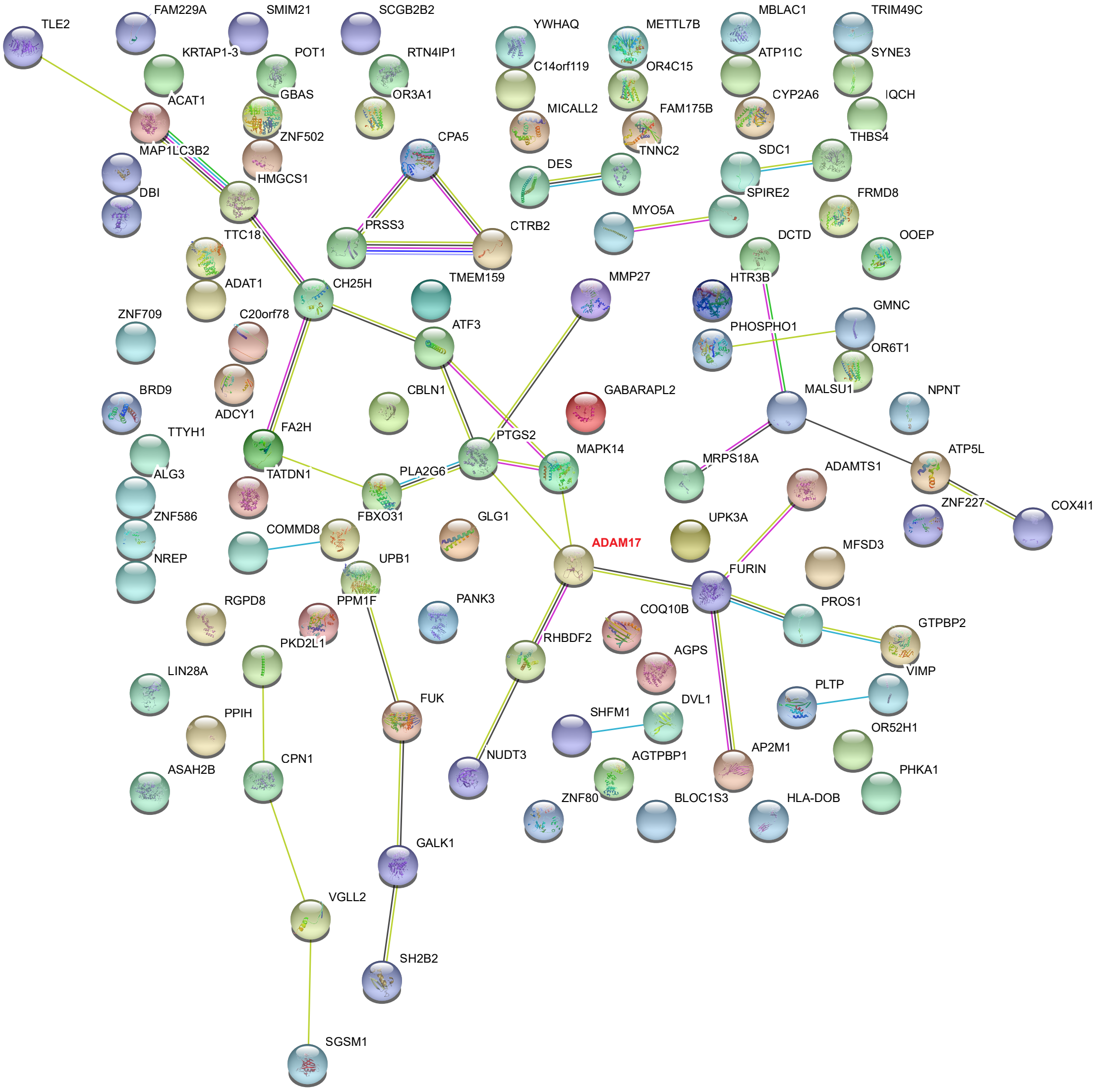

Figure S5. STRING analysis of the hits in the non-shedding population of the CRISPR/Cas9 genome-wide screen (related to Fig. 4). STRING analysis that depicts potential protein-protein interactions within the group of proteins that were scored as hits, as defined by an enrichment of their mean LFC >2. ADAM17 is highlighted in red.

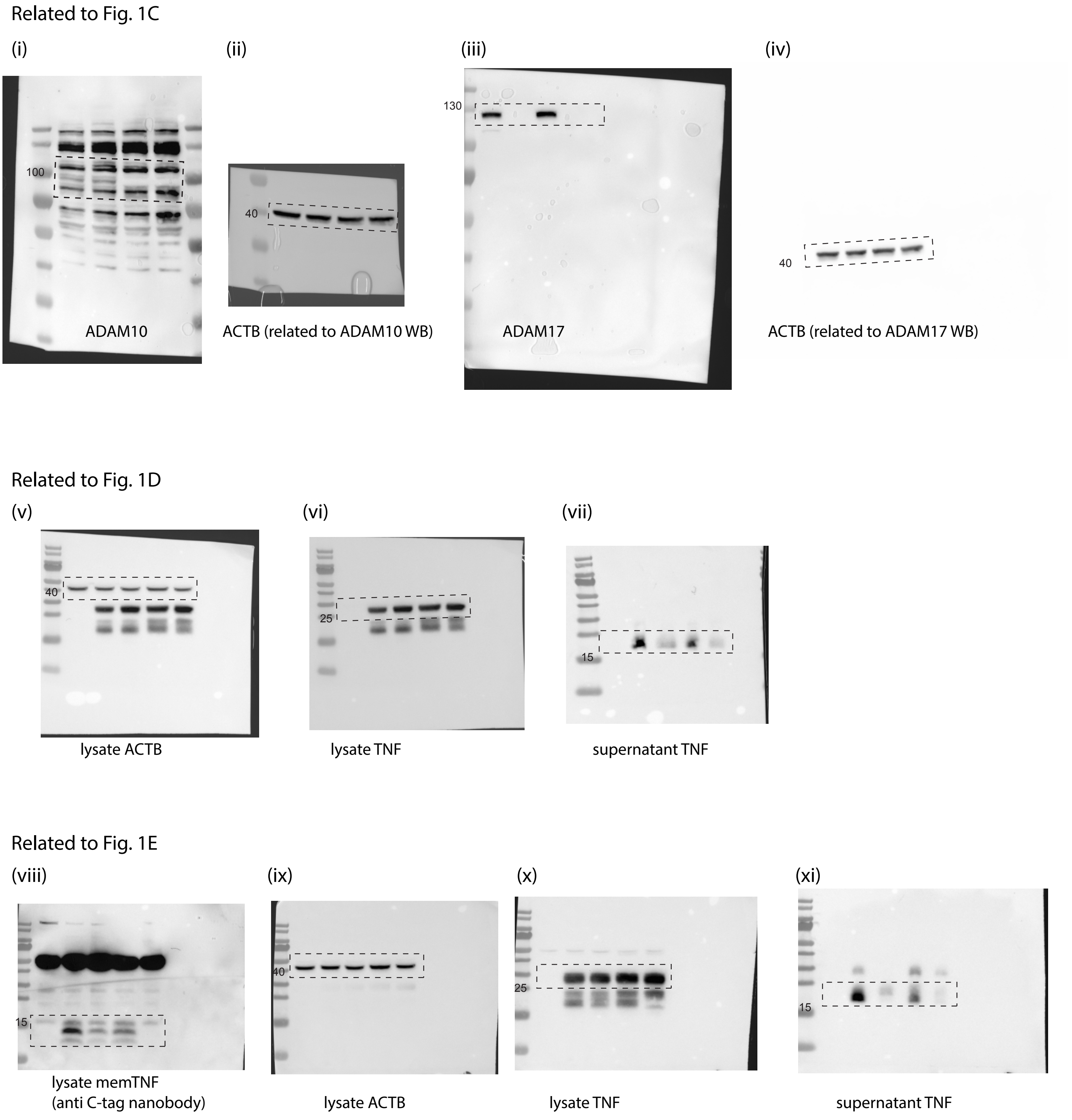

Figure S6. Uncropped immunoblot images (related to Fig. 1). Uncropped immunoblot images of Fig. 1C, D and E are shown. The dashed lines indicate the cropping margins. In order to display the size marker, a merged image of the image derived from the chemiluminescent signal and the image derived from the white-light epi-illumination signal is depicted (except for image (iv)). Of note, in Fig. 1C, D and E only the image derived from the chemiluminescent signal is shown.
